# Characterizing neurocognitive impairment in young people with major depression: state, trait, or scar?

**DOI:** 10.1002/brb3.527

**Published:** 2016-07-21

**Authors:** Kelly Allott, Caroline A. Fisher, Gunther Paul Amminger, Joanne Goodall, Sarah Hetrick

**Affiliations:** ^1^ OrygenThe National Centre of Excellence in Youth Mental HealthParkvilleVictoriaAustralia; ^2^ The Centre for Youth Mental HealthThe University of MelbourneParkvilleVictoriaAustralia; ^3^ The Melbourne ClinicRichmondMelbourneAustralia; ^4^ Royal Melbourne HospitalParkvilleMelbourneAustralia

**Keywords:** adolescence, cognition, deficits, early intervention, major depressive disorder, neuropsychological, young adulthood, youth

## Abstract

**Background:**

Major depressive disorder (MDD) affects a quarter of adolescents and young adults and is associated with the greatest global burden of disease in this population. There is a growing literature, mostly in adults, showing that significant neurocognitive impairments are common in MDD. It remains unclear whether these impairments are pre‐existing trait markers of MDD, state‐related impairments that fluctuate with depressive symptoms, or ‘scar’ impairments that worsen with illness progression. The aim of this study is to provide a conceptual framework for understanding MDD and neurocognitive impairment in adolescence and young adulthood (ages 12–25 years).

**Method:**

Examination of the evidence for neurocognitive deficits as trait, state, and scar features of MDD according to different study designs (family studies, premorbid studies, current depression, remitted depression, and longitudinal studies with repeated assessment) was conducted.

**Results:**

The few premorbid and family studies conducted in youth provide equivocal evidence for neurocognitive impairments as trait markers of MDD. The presence of state‐based neurocognitive impairment remains unclear as evidence comes mostly from cross‐sectional studies. There are a limited, but growing number of longitudinal studies with repeated neurocognitive assessment in youth. Studies that examined neurocognition prior to the onset of MDD and with long‐term follow‐up provide tentative evidence for neurocognitive scarring.

**Conclusion:**

Neurocognitive impairment is a feature of MDD in adolescents and young adults. To better understand the nature, timing, and pattern of impairment, longitudinal studies that examine neurocognition before and after the development of full‐threshold MDD, including following recurrence are needed. This knowledge will have important implications for mechanisms, prevention, and treatment of MDD in youth.

## 1 Introduction

Major depressive disorder (MDD) is a common psychiatric disorder, which peaks in onset during adolescence and young adulthood (Kessler et al., 2005). By the age of 19 years, up to one in four young people in Western countries will have experienced a major depressive episode (Australian Institute of Health and Welfare, 2011; Fergusson, Boden, & Horwood, 2007; Lewinsohn, Rohde, & Seeley, 1998; Rohde et al., 2013). Adolescence and young adulthood represents a critical period due to the dynamic neurological and neurocognitive developmental processes occurring during this phase of life (Casey, Getz, & Galvan, 2008; Giedd, 2004; Paus, Keshavan, & Giedd, 2008) and because the formation and maintenance of social and intimate relationships and educational and vocational attainment are at the forefront. Major depression tends to be recurrent, particularly for those with a young age of onset (Birmaher et al., 2004; Fombonne et al., 2001). With each episode that is experienced there is an increased risk of recurrence (Lewinsohn et al., 2000; Solomon et al., 2000). Over the course of repeated episodes there is also a worsening pattern (Kessing, Hansen, & Andersen, 2004), with more frequent recurrence, greater severity, and more resistance to initially effective treatments (Kendler, Thornton, & Gardner, 2000). Furthermore, MDD confers significant risk for suicidal ideation, suicide attempts, and completed suicide (Cash & Bridge, 2009; Foley, Goldston, Costello, & Angold, 2006; Harrington et al., 1994; Rao, Weissman, Martin, & Hammond, 1993; Weissman et al., 1999). Thus, the onset of MDD during adolescence/young adulthood is not only contemporaneously disruptive and potentially life threatening, if not adequately treated, it can be associated with lifelong impairment and disability (Fergusson & Woodward, 2002; Fergusson et al., 2007; Weissman et al., 1999). Indeed, because of the marked prevalence and impact of MDD, it accounts for the greatest global burden of disease in young adults (Gore et al., 2011) and is projected by the World Health Organisation (WHO) to be the leading cause of disability globally by 2030 (Mathers, Fat, & Boerma, 2008). Thus, identifying markers of risk, illness progression and barriers to recovery, and tailoring evidence‐based interventions accordingly to the clinical presentation are critical for effective early intervention in MDD.

Neurocognitive impairments, such as poor concentration and memory, slowed speed of information processing, and difficulties organizing one's thinking (i.e., executive dysfunction), are a central feature of many psychiatric conditions, including MDD (Millan et al., 2012). Empirical studies on neurocognition in MDD have steadily increased over recent decades and have provided robust evidence for widespread neurocognitive impairments in MDD samples relative to healthy controls (Douglas & Porter, 2009; Rock, Roiser, Riedel, & Blackwell, 2014). Neurocognitive impairments in MDD have been associated both with poorer response to treatment (Bruder et al., 2014; Gallagher et al., 2007; Gordon et al., 2015; Potter et al., 2004) as well as poorer social and vocational functioning and greater disability in adults (Baune et al., 2010; Jaeger, Berns, Uzelac, & Davis‐Conway, 2006; Woo et al., 2016) as well as young people (Lee et al., 2013) with MDD.

More recently, the field has moved toward investigating the precise nature and mechanisms of neurocognitive impairment in MDD, particularly in light of the possible progressive nature of the illness (Hasselbalch, Knorr, & Kessing, 2011). Specifically, an important question is whether neurocognitive impairments in MDD are pre‐existing trait/vulnerability markers, state‐related impairments that fluctuate with depressive symptoms, and/or ‘scar’ impairments that remain during periods of remission and worsen with illness progression. It is essential to understand the nature and source of neurocognitive impairments in MDD because this will advance current etiological theories and guide the specific prevention or intervention strategies that might be used (Allott et al., 2013).

The aim of this study is to provide a conceptual framework for understanding neurocognitive impairment in MDD and to examine the current evidence for neurocognitive deficits as trait, state, and/or scar features of MDD. Findings and discussion will be organized according to the various study designs that inform the trait, state, and/or scar hypotheses of neurocognitive impairment. There will be a specific focus on studies of adolescence and young adulthood (defined as ages 12–25 years) for two reasons. First, this a highly vulnerable developmental period of life and risk period for the first onset of MDD. Second, data gathered on youth are less confounded by factors associated with chronic MDD that may impact neurocognitive function, such as long‐term use of medication and poly‐pharmacy, substance use, or other psychiatric comorbidity, hospitalizations, electroconvulsive therapy, etc. Nevertheless, given the emerging nature of this field in youth cohorts, published reviews of studies in adults will also be included as a point of comparison and where findings in youth are lacking. This is not intended to be a systematic review of the literature on neurocognition in MDD, which is beyond the scope of this study. The overarching aim is to advance current theories of the origin and evolution of neurocognitive impairment in youth MDD. It is also hoped that this study will provide guidance as to areas for further research on neurocognition in youth MDD.

## 2 Conceptual and Study Design Framework for Understanding Neurocognitive Impairment in MDD

Neurocognitive impairment in MDD may be broadly interpreted as trait‐, state‐, or scar‐based impairment. It is important to note that trait, state, and scar patterns of neurocognitive impairment may co‐occur and different study designs may provide evidence for more than one of these mechanisms. Each of these patterns of impairment, including how different study designs can help to differentiate them, is described in more detail below.

### 2.1 Trait neurocognitive impairment

For a characteristic to be considered a possible trait or risk marker, it must be clearly associated with the illness in question (i.e., predicts later onset of illness), but also independent of clinical state. Specifically, it must be a stable persistent feature that is detectable prior to illness onset and also present during periods of symptomatic remission (Gottesman & Gould, 2003). Evidence for trait neurocognitive impairment comes from studies that investigate neurocognitive functioning of individuals (as early as childhood) before they develop full‐threshold first‐episode MDD. Evidence may also come from studies of remitted MDD samples, although cross‐sectional studies of remitted MDD cannot differentiate trait from scar effects. Only longitudinal studies assessing neurocognition before and after a major depressive episode can disentangle trait from scar impairments.

Trait impairments may occur through biological (heritable or nonheritable) or environmental mechanisms. For example, prenatal or early life stress might cause persistent neurocognitive deficits in childhood that are associated with later risk for MDD. For neurocognitive impairment to be considered a heritable trait putatively linked to a gene or genes (also referred to as an endophenotype), it should be more frequently present or more severe in relatives unaffected by MDD than in the general population, while still being more frequently present or severe in the affected versus unaffected family members (Gottesman & Gould, 2003). Thus, family studies showing similar, although attenuated impairments in unaffected relatives of individuals with MDD would provide evidence for neurocognitive deficits as heritable traits (endophenotypes) of MDD. If neurocognitive impairments are found to be traits of MDD, they could be used as early markers for identifying those at risk of developing MDD, including identifying specific thinking and coping styles related to depressive symptoms (e.g., rumination) (Han et al., 2016; Snyder, 2013). Trait neurocognitive impairments, therefore, may be an important focus of preventive interventions (Hetrick et al., 2008).

### 2.2 Scar neurocognitive impairment

Progressive decline or attenuated development in neurocognitive functioning associated with the onset and progression of MDD would indicate scar‐related neurocognitive impairment. As implied by the term ‘scar’, neurocognitive functioning would be expected to worsen with increased severity and chronicity of illness (e.g., number of depressive episodes, duration of illness, etc.). In adolescents and young adults who are still undergoing significant neurological (particularly fronto‐temporal) and neurocognitive development, scar neurocognitive impairment may not necessarily manifest as a decline or progressive worsening course. In this instance, it may be that the adolescent still shows improved neurocognitive performance in line with ongoing development during this stage of life, but their development might be *attenuated* relative healthy peers (Anderson, Northam, Hendy, & Wrennall, 2001; Vijayakumar et al., 2016). The resulting neurocognitive pattern may then reflect a developmental ‘lag’ or even ‘arrest’, instead of decline *per se*. Furthermore, because of the dynamic phase of development during adolescence and young adulthood, impairments in neurocognition may not be proximally evident in relation to MDD and may only begin to emerge after passing through this neurodevelopmental period (Anderson et al., 2001). Cross‐sectional studies that compare individuals with less severe/chronic MDD with more severe/chronic MDD, or that investigate the relationship between the degree of neurocognitive impairment and number of previous depressive episodes or length of illness in people who are in remission from MDD, may provide partial support for the scarring hypothesis. However, repeated neurocognitive testing of the same individuals via longitudinal designs provides the most rigorous evidence for scar‐like effects. In adolescents and young adults, follow‐up periods would need to span several years in order to capture the interaction between prolonged neurodevelopment, neurocognitive functioning, and MDD.

Possible causes of progressive neurocognitive scarring include pathways regulating inflammation, oxidative repair (Galecki et al., 2015), apoptosis and neurogenesis (Lee, Reif, & Schmitt, 2013), as well as dysregulation of the hypothalamic–pituitary–adrenal axis (Vreeburg et al., 2009). If neurocognitive scarring is a possible consequence of MDD, early identification through regular neurocognitive assessments in combination with routine blood biomarkers may be indicated. Accordingly, psychological and biological interventions such as cognitive remediation therapy or specifically targeting potential pathomechanisms (e.g., inflammation) may be important components to the treatment armamentarium.

### 2.3 State neurocognitive impairment

State‐based neurocognitive impairments are evident if they co‐occur with depressive symptomatology and increase or decrease with the exacerbation or resolution of depressive symptomatology. They can also be expected to be more severe with greater symptom severity and may occur over and above existing trait or scar impairments. Cross‐sectional studies of neurocognitive functioning of individuals with current MDD are the most common design for examining this, but are particularly nonspecific because they cannot distinguish state‐like from trait‐like neurocognitive deficits, or can they completely rule out scar‐like effects. Better evidence for state‐related impairment would come from longitudinal studies showing that neurocognition improves following depressive episode remission or negative findings from adequately powered cross‐sectional studies of neurocognition in remitted MDD. Evidence could also include experimental findings showing that induced depressed mood produces poorer neurocognition, or studies using experience sampling methodology that can examine the temporal relationship between subjective cognitive and mood changes.^1^

State‐related neurocognitive impairment may be caused through alterations in neurotransmitter levels in the neurotransmitter systems that govern both mood and neurocognitive skills. By definition, many clinical symptoms of depression directly relate to neurocognitive function, including a diminished ability to think or concentrate, indecisiveness, sleep deprivation, and fatigue (American Psychiatric Association, 2013). Additionally, feelings of hopelessness and reduced motivation are likely to impair effort and performance on neurocognitively demanding tasks. If state‐related neurocognitive impairments are a feature of MDD, then clinicians may need to adapt their therapy to ensure individuals derive benefit. For example, in the case of diminished ability to concentrate, clinicians may have shorter therapy sessions or focus more on behavioral activation rather than cognitive restructuring when delivering cognitive‐behavior therapy. Neurocognitive functioning might also form part of the psychoeducation provided to clients, including the fact that neurocognitive impairments may contribute to poorer functioning while unwell. Additionally, neurocognitive impairment might also be used as a marker of treatment response (Gallagher et al., 2007).

**Figure 1:**
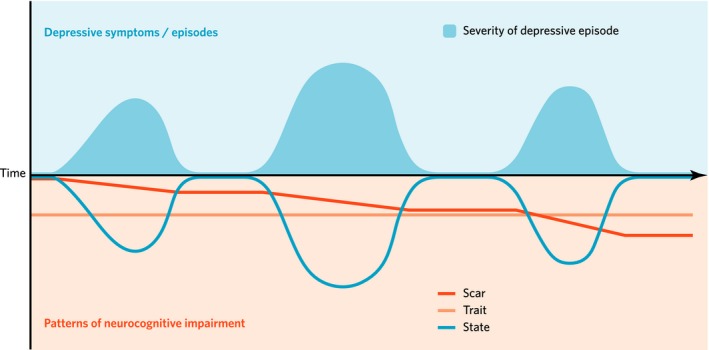
Model of trait, state and scar patterns of neurocognitive impairment in relation to major depressive disorder

Figure 1 illustrates the conceptual model of trait, state, and scar patterns of neurocognitive dysfunction in relation to MDD. It is important to note that the model depicted in Figure 1 does not specifically include attenuated neurocognitive development as described earlier in relation to scarring in the context of neurodevelopmental processes. The aim of the figure is to simplistically illustrate the concepts of trait, state, and scar impairment. Table 1 outlines the various study designs that can provide evidence for trait, state, and scar patterns of neurocognitive impairment in MDD. Evidence according to these different study designs is examined in the following sections.

**Table 1:** Evidence for trait, state, and scar patterns of neurocognitive impairment in MDD according to different study designs

Study design	Trait	State	Scar
Family Studies	Deficits present	No deficits present	No deficits present
Premorbid Studies	Deficits present	No deficits present	No deficits present
Current Depression	Deficits present	Deficits present	Deficits present
Remitted Depression	Deficits present	No deficits present	Deficits present
Longitudinal Studies	Similar deficits are present premorbidly and during remission.	Deficits co‐occur with depressive symptomatology and increase or decrease with exacerbation or resolution of depressive symptomatology.	Deficits are present after remission from a depressive episode and are worse than before the episode. Deficits progress with increased severity and chronicity of MDD.

## 3 Evidence of Pattern of Neurocognitive Impairment in MDD from Different Study Designs

### 3.1 Family studies

Having a family member with MDD places an individual at increased risk of the disorder; neurocognitive impairment may be a heritable trait marker for MDD. However, there are relatively few studies that have examined the neurocognitive functioning of unaffected first‐degree relatives of people with MDD in general, let alone in young people. A study of unaffected children (mean age 9 years) (Micco et al., 2009) and another of unaffected adolescent offspring (mean age of 16) (Klimes‐Dougan, Ronsaville, Wiggs, & Martinez, 2006) of mothers with MDD found no significant impairments in neurocognitive functioning (attention, processing speed, memory, executive functioning) compared to healthy controls. However, on the contrary, two relatively recent studies have suggested that having a parent with depressive pathology confers risk for executive functioning impairments in asymptomatic offspring. One study tested offspring with a mean age 16 who had a parent with MDD (Belleau et al., 2013), and the other examined the relationship between mothers' self‐reported depressive symptoms prospectively assessed over 4 years (when their children were aged 2–6 years) and the executive functioning of their children at age 6 (Hughes, Roman, Hart, & Ensor, 2013). Both studies showed a relationship between parental depressive pathology and neurocognitive impairments in their nondepressed children. However, it cannot be determined whether these impairments are related to biological or environmental factors or a combination of both.

Studies of twins raised in the same environment provide a more powerful avenue for examining this issue (Hsu et al., 2014). To date twin studies have only been conducted in adults. An early study compared the neurocognitive performance of unaffected twins from monozygotic and dizygotic twin pairs, where either their co‐twin was affected by MDD (placing them at high risk for MDD) or their co‐twin was also unaffected (placing them at low risk for MDD) (mean age ranged from 38 to 48 years across the twin combinations) (Christensen, Kyvik, & Kessing, 2006). After controlling for demographic and clinical variables, the healthy twins with a co‐twin affected by MDD showed impairments in attention, working memory, executive function, language processing, and memory compared with those who did not have a twin affected by MDD, supporting the notion of genetic liability for neurocognitive impairment in MDD (Christensen et al., 2006). Similarly, a more recent study of adult (mean age 45 years) monozygotic twins discordant for MDD found that after controlling for age and gender there was evidence for impairments in attention and general knowledge in both the affected and unaffected twins, compared to twin pairs without a history of MDD (Hsu et al., 2014). Furthermore, probands with early‐onset depression (<18 years of age) performed more poorly on a visuoconstructional task than their unaffected co‐twins. Current level of depressive symptoms was unrelated to neurocognitive performance (Hsu et al., 2014). Findings from these studies provide some evidence for neurocognitive impairment as a heritable trait in MDD, but the latter study also suggests that depression during adolescence may leave a neurocognitive scar independent of genetic vulnerability (Hsu et al., 2014).

### 3.2 Premorbid studies

Premorbid studies include large birth or military conscription cohort studies that have examined the neurocognitive functioning of generally healthy individuals (children or adolescents) before they experience the first onset of psychiatric disorder, including MDD. Although these studies involve longitudinal designs, they are included in this section because neurocognitive functioning is only examined at one initial time point and change in neurocognitive functioning cannot be determined. Most of these studies have examined IQ rather than specific neurocognitive domains. Lower premorbid IQ has been associated with a mildly increased risk of a subsequent diagnosis of severe depression requiring hospital admission (OR = 1.22) (Zammit et al., 2004) and an ICD‐8 diagnosis of ‘depressive neurosis’ (OR = 1.08) (David et al., 2008). Another study showed that for each standard deviation increase in childhood IQ there was a 23% reduction in the odds of having an adult diagnosis of major depressive episode, whereas lower IQ was associated with a greater risk of persistent depression between the ages of 18 and 32 (Koenen et al., 2009). Finally, Glaser et al. (2011) investigated the association between IQ assessed at age 8 years with self‐reported depressive symptoms at 11, 13, 14, and 17 years. Interestingly, they found that there was an association between childhood IQ and depressive symptoms in adolescence, but that the direction of the relationship varied according to age and pubertal stage (assessed using a scale of pubic hair and in females also breast development). Specifically, lower IQ was associated with higher depressive symptoms at age 11, but this association was reversed at ages 13 and 14, such that higher IQ was associated with higher depressive symptoms. This latter association persisted for 17‐year‐old males, but not females. These findings were broadly replicated by a similar recent longitudinal study (Weeks et al., 2014). The authors speculated that these complex findings may be associated with the onset and offset of pubertal changes, which are known to be associated with IQ (Ramsden et al., 2011) and to differ for females and males (Glaser et al., 2011; Weeks et al., 2014). Although MDD was not the outcome of either study, the findings highlight age, gender, and developmental factors (such as puberty) as important considerations when investigating neurocognitive functioning in the context of emerging and full‐threshold MDD.

Whether specific neurocognitive deficits (as opposed to general IQ) are evident before the onset of a first episode of MDD, including during ‘prodrome’ periods, remains an open area of investigation, as defining the risk syndrome or prodromal phase of MDD has been a challenge for the field (Hetrick et al., 2008). A prospective birth cohort study that assessed individuals from the age of 3 found that there was no difference between healthy controls and individuals with a diagnosis of ‘depression or anxiety disorder’ at age 26, in their motor, expressive, and receptive language abilities assessed at ages 3, 5, 7, and 9 (Cannon et al., 2002). However, individuals who were diagnosed with ‘depression or anxiety disorder’ at age 26 displayed significant deficits in psychomotor speed and attention at age 13 (Cannon et al., 2006). A challenge in interpreting these findings is that it remains unclear when participants first started experiencing depressive (or anxiety) pathology and it is possible that subsyndromal depressive or anxiety symptoms were present during early adolescence. In a study of 192 young adolescent school students (mean age 12.4, range 9–15 years) Evans, Kouros, Samanez‐Larkin, and Garber (2016) investigated the relationship between executive functioning (working memory and cognitive flexibility) measured at time 1 and coping and depressive symptoms measured concurrently and again at 4‐month follow‐up. Poorer executive functioning at time 1 was associated with higher depressive symptoms at baseline. While controlling for time 1 depressive symptoms, age, and IQ, executive functioning at time 1 was predictive of an increase in depressive symptoms at time 2 (4 months). Coping partially mediated the relationship between executive functioning and depressive symptoms, indicating that executive functioning impairment may pose a risk for MDD through its association with coping style. A similar study by (Han et al. (2016) examined the concurrent and 2‐year prospective association between time 1 executive functioning and times 1 and 2 depressive symptoms (as well as other psychopathology) in a community sample of 220 adolescents (mean age 13.7; range 11–16 years). The sample was ‘enriched’ with respect to internalizing and externalizing problems. There was no association between time 1 executive functioning and concurrent self‐reported depressive symptoms, but mother‐reported symptoms of depression in their offspring was associated with executive functioning at time 1 (Han et al., 2016). However, while controlling for IQ, gender, and age, executive functioning at time 1 was not associated with self‐ or mother‐reported depressive pathology at 2‐year follow‐up (Han et al., 2016). An earlier study found no premorbid impairment in executive functioning, psychomotor speed, and attention in adolescents (mean age 14.8 years) who were later diagnosed with MDD in early adulthood (mean age of 21.7 years) (Meyer et al., 2004).

Whereas lower IQ appears to have an association with increased risk for MDD, the evidence regarding specific neurocognitive domains, particularly executive functioning impairments, as risk markers for MDD is limited and inconclusive.

### 3.3 Current depression

Many cross‐sectional studies have examined neurocognitive functioning in currently depressed individuals, with numerous reviews having been conducted, showing evidence for small‐to‐moderate impairments in several neurocognitive domains (Austin, Mitchell, & Goodwin, 2001; McDermott & Ebmeier, 2009; Rock et al., 2014; Snyder, 2013). However, most of this research has focused on middle‐aged or older adults with a history of multiple episodes. Investigating neurocognitive functioning during a first episode of MDD has the advantage of characterizing impairments that are independent of possible ‘scarring’ effects associated with a relapsing/multiepisode or chronic course of illness. A systematic review and meta‐analysis by Lee, Hermens, Porter, and Redoblado‐Hodge (2012) of 13 studies of neurocognitive functioning in currently depressed or remitted first‐episode MDD diagnosed in adulthood (mean age 39 years) found significant small‐to‐medium impairments in psychomotor speed (Hedges' *g *=* *0.48), attention (Hedges' *g *=* *0.36), visual learning and memory (Hedges' *g *=* *0.53), and several domains of executive functioning (attentional switching, cognitive flexibility, verbal fluency: Hedges' *g *=* *0.22–0.59) (Lee et al., 2012). No impairments were found in working memory and verbal learning and memory (Lee et al., 2012). Psychomotor speed, working memory, and general memory functioning were moderated by inpatient and remission status (Lee et al., 2012), suggesting that some domains of neurocognitive functioning in MDD may be state driven. Antidepressant medication use was associated with poorer verbal learning and memory, but better cognitive flexibility, underlining medication status as an important covariate. Age was also a moderating variable, with older age being associated with poorer neurocognitive performance in first‐episode MDD (Lee et al., 2012). This suggests that there may be differences in the etiology and pathophysiology of MDD depending on the age it first presents. The review by Lee et al. (2012) excluded studies of people under 18 years of age, thus overall results and those specifically regarding age, cannot be generalized to adolescents. Furthermore, despite Lee et al.'s explicit inclusion of studies of patients with a first episode of MDD, it is possible that in some cases previous depressive episodes may have been undetected, as it has been found that by 19 years of age a quarter of young people have already had at least one episode (Lewinsohn et al., 1998).

Recently, Baune, Fuhr, Air, and Hering (2014) reviewed studies of neurocognitive functioning in young people aged 12–25 years with MDD. Seven cross‐sectional studies were included; it is important to note that only two of these involved adolescents with first‐episode MDD. The most consistent neurocognitive deficits were in working memory and psychomotor speed, although there was equivocal evidence in executive functioning, verbal fluency, and visual memory, and no evidence for impairment in attention, verbal learning, and memory (Baune et al., 2014). One of the first‐episode studies examined neurocognitive functioning in medication‐naïve adolescents (aged 12–17 years) with current MDD relative to healthy controls (Klimkeit et al., 2011). They found selective impairments in processing speed and working memory, but no impairment in executive functioning. In the other first‐episode study, the participants appeared to be in remission (Kyte, Goodyer, & Sahakian, 2005); thus, results are presented in the following section. An additional cross‐sectional study by Maalouf et al. (2011) (not included in the Baune et al. (2014) review) aimed to examine the state versus trait question by comparing adolescents (mean age 15 years) with either current (*n *= 20) or remitted MDD (*n *= 20) with healthy controls (*n *= 17) on tasks of executive function, sustained attention, and short‐term memory (Maalouf et al., 2011). Significant executive functioning impairments were evident in the currently depressed sample, but not in those in remission (Maalouf et al., 2011). Although these findings suggest that impairments are most likely state related, the sample size was small and cross‐sectional comparison of independent groups does not rule out the possibility of pre‐existing trait‐related impairments in the currently depressed group, or of the later emergence of ‘scarring’ in the remitted adolescent group. As discussed previously, in addition to longitudinal studies, large studies of remitted MDD that demonstrate intact neurocognitive functioning would provide better support for the state‐related model.

### 3.4 Remitted depression

Several cross‐sectional studies have assessed neurocognitive functioning in adults in remission from MDD (usually defined by a cut‐off score on clinician‐rated scales maintained over a specified duration). A systematic review and meta‐analysis of 27 studies by Bora, Harrison, Yucel, and Pantelis (2013)) found a broad range of neurocognitive impairments of small‐to‐medium effect size (*d *=* *0.39–0.59) in adults in remission from MDD relative to healthy controls (Bora et al., 2013). This review included a subanalysis of early‐ versus late‐adult onset MDD samples, and found that late‐onset MDD (onset after age 60) was associated with significantly larger neurocognitive impairments compared with early‐onset MDD, suggesting different etiological mechanisms possibly involving vascular and neurodegenerative factors in late‐onset MDD (Bora et al., 2013). As the ‘early‐onset’ samples included people up to the age of 59 years, specific interpretations regarding the impact of MDD in adolescence or early adulthood cannot be made. Nevertheless, their meta‐regression analysis examining the impact of early‐ and late‐onset showed that the number of previous episodes and duration of illness had no significant influence on the degree of neurocognitive deficits observed in euthymic MDD patients (Bora et al., 2013). This finding does not support a progressive ‘scarring’ pattern of neurocognitive impairment associated with illness chronicity.

A second systematic review and meta‐analysis by Rock et al. (2014) focused only on studies that used the Cambridge Neuropsychological Test Automated Battery (CANTAB) to assess neurocognition; six studies of people in remission from MDD were included. In this meta‐analysis, remitted MDD was associated with significant moderate deficits compared to healthy controls in executive functioning (*d *=* *0.53–0.61) and attention (*d *=* *0.52). The authors did not examine whether there was a relationship between neurocognitive impairments and illness characteristics such as number of episodes, age of onset, and depression severity.

Research involving youth in remission from MDD remains limited. Of the included studies in the Bora et al. (2013) meta‐analysis, the lowest mean age of participants was 34 years, with no studies focusing on youth specifically, and in the Rock et al. (2014) meta‐analysis only one study involved young people (Maalouf et al., 2011). This was the study by Maalouf et al. (2011) described earlier with their finding of no evidence for impairments during remission in 20 people aged 15 years on average. In contrast, Kyte et al. (2005) found that adolescents (mean age 15 years; *n *= 30) who had a diagnosis of first‐episode MDD in the previous year and whose current mean mood ratings were not significantly different from *n *= 49 healthy controls (suggesting many were likely in remission), displayed impulsivity on a decision‐making task. Medication use and previous depression severity did not influence the findings (Kyte et al., 2005). Another study of 42 young adults (mean age 21.3 years) who had been in remission from recurrent MDD for at least 1 month found significant moderate‐to‐large impairments in immediate verbal memory, processing speed, and executive functioning relative to healthy controls (*n *= 33) (Smith, Muir, & Blackwood, 2006). Without data on neurocognitive functioning prior to MDD, it remains unclear whether the findings of the latter two studies reflect trait‐ and/or scar‐related impairments. Furthermore, residual (still resolving) state‐based neurocognitive effects may have been present in these samples.

### 3.5 Longitudinal studies with repeated neurocognitive assessment

A systematic review of 30 studies by Douglas and Porter (2009) that examined the longitudinal course of neurocognitive functioning and its relationship to symptomatology in adults with MDD revealed that improvements in verbal memory, verbal fluency, and psychomotor speed were closely related to improvements in mood, whereas attention and executive functions remained impaired despite clinical status—suggestive of trait‐ and also possibly scar‐based impairment (Douglas & Porter, 2009). However, a drawback of many of the studies included in the Douglas and Porter (2009) review was the lack of a matched healthy comparison group to clearly characterize the degree of normal versus abnormal neurocognitive change. The relatively short follow‐up assessments (weeks to months) in most studies raises the possibility that the neurocognitive domains showing improvement may have been those most susceptible to practice effects. Furthermore, none of the studies included young people who were early in the course of MDD.

The earlier in the illness course that change in neurocognition is examined, the ‘cleaner’ and more interpretable the findings are with respect to trait versus state versus scar impairments. Since the Douglas and Porter review, a growing number of longitudinal studies involving repeated assessment of neurocognitive functioning in youth with MDD have emerged. Peters et al. (2015) investigated verbal fluency, processing speed, conceptual reasoning, set shifting, and cognitive control in youth aged 18–23 years (*n *=* *62) who had a history of 1–3 depressive episodes, but were in remission from MDD and not taking medication, compared to healthy controls. They examined whether any impairments observed during remission resolved, remained stable, or got worse at follow‐up assessment 3–15 weeks later (Peters et al., 2015). Only a single small‐to‐moderate deficit (*d *=* *0.38) in cognitive control (the ability to regulate and inhibit responding based on previously presented information) was observed in remitted youth at time 1; this impairment remained stable at time 2. The impairment in cognitive control was unrelated to residual depressive or anxiety symptoms, number of prior depressive episodes, age at onset, number of hospitalizations, longest episode duration, years since last episode, or ever being prescribed medication (Peters et al., 2015).

An earlier prospective study by Schmid and Hammar (2013) assessed the neurocognitive functioning of slightly older young adults (mean age 26 years; *n *=* *28) during their first episode of MDD and again at 12 months follow‐up, compared to a healthy control group (Schmid & Hammar, 2013). Similar to Petersen et al., there was again evidence for persistent executive functioning impairments (specifically in inhibition, switching, and semantic fluency), despite a reduction in depressive symptoms over the 12‐month period. Furthermore, Schmid and Hammar (2013) found that participants with poorer performance in inhibition/switching in the acute phase of illness (time 1) had a greater tendency to experience a relapse within the first year (Schmid & Hammar, 2013). Another recent study administered a range of subtests from the CANTAB to adolescents aged 13–18 years (*n *=* *13) who had recently recovered from a major depressive episode and again at 2‐month follow‐up, compared to healthy controls (Bloch et al., 2015). They found that while attention improved over the 2‐month period, suggestive of a state‐related impairment, stable visual working memory impairments were evident over the follow‐up period (Bloch et al., 2015). Together the findings of these longitudinal studies are suggestive of mild trait‐related neurocognitive impairments in youth with MDD because impairments were relatively stable and unrelated to illness severity, and in one study predicted relapse (Schmid & Hammar, 2013). However, because neurocognitive functioning prior to the onset of MDD was unknown in all of these studies and the follow‐up periods were relatively short, it is not clear when the neurocognitive impairments emerged and thus, the direction of the relationship between neurocognitive impairment and depression onset. Assessment of premorbid neurocognition and longer follow‐up periods including the impact of relapse would be more informative with respect to possible scar‐related effects. A longitudinal study conducted by Beaujean, Parker, and Qiu (2013) used path modeling to examine the causal relationship between depressive symptoms and vocabulary in a large cohort (*n *=* *14,322) of adolescents at baseline (mean age of 15 years) and then 8 years later in young adulthood (mean age 22 years). They found that there was a cross‐sectional relationship between depressive symptoms and vocabulary at both time points. While controlling for baseline depression, vocabulary, and other covariates (gender, ethnicity, English fluency, parental education), the level of depressive symptoms in adolescence was significantly associated with vocabulary ability in early adulthood, but adolescent vocabulary ability was not related to early adulthood depression levels. Moreover, after controlling for adolescent levels of depression and vocabulary, the vocabulary–depression relationship disappeared in adulthood. These intriguing findings are in contrast to the previous studies as they provide support for the scar hypothesis.

A recent prospective study by Vijayakumar et al. (2016) examined the relationship between cognitive control and onset of MDD during early and late adolescence. Young adolescents (*N *=* *165; mean age 12.7 years) with no current or past history of MDD completed a cognitive control task at time 1 and again 4 years later (time 2; mean age 16.5 years). Assessment of MDD was conducted at time 1 and 2, and again 2 years later (time 3; mean age 18.9 years). IQ (assessed at time 1) was unrelated to the onset of MDD in early or late adolescence. However, change in cognitive control from time 1 to time 2 differed depending on the timing of MDD onset. Specifically, cognitive control improved in accordance with normal development in adolescents who either did not develop MDD over the three time points (*n *= 122) or who developed MDD in late adolescence (time 2 to 3; *n *= 20) (Vijayakumar et al., 2016). In contrast, an ‘arrest’ in development of cognitive control was observed in those who experienced MDD during early adolescence (time 1 to 2; *n *= 23) (Vijayakumar et al., 2016). All three groups performed similarly on the cognitive control task at time 1; however, as the timing and length of acute depression in the early‐onset MDD group was not reported, it remains unclear whether abnormal development of cognitive control was of trait‐ or scar‐based origin. The fact that there was normal development of cognitive control in the late‐onset MDD group lends support to the scarring hypothesis, but no firm conclusions can be drawn without assessment of cognitive control at time 3, which did not occur in the study. A limitation of the Beaujean et al. (2013) and Vijayakumar et al. (2016) studies is that only one neurocognitive domain was examined at two time points, thus future longitudinal studies involving assessment of a broader range of neurocognitive functions that are assessed at multiple times should provide further clarification.

## 4 Discussion and Future Directions

We have presented evidence from various study designs on neurocognitive functioning in MDD, with a particular focus on adolescents and young adults, to shed light on whether neurocognitive deficits reflect trait, state, and/or scar features of the illness. While studies of adults have previously dominated this field of research, there has been recent growth of studies in youth, with the recognition that this is a critical developmental period of life that is associated with heightened risk for the onset of MDD.

Given the evidence presented from the various study designs, what can be said about the nature of neurocognitive impairment in youth with MDD? The limited premorbid and family studies provide equivocal evidence for neurocognitive impairments being trait markers of MDD. Of the few family studies conducted, two studies showed neurocognitive (specifically executive functioning) deficits in unaffected offspring of mothers with depression (Belleau et al., 2013; Hughes et al., 2013) and two did not (Klimes‐Dougan et al., 2006; Micco et al., 2009). Unaffected twins of adult twin pairs discordant for MDD were found to be impaired in various neurocognitive domains (Christensen et al., 2006; Hsu et al., 2014), but it was not clear when the affected adult twins first experienced MDD, thus developmental factors could not be considered. To our knowledge there are no twin studies conducted in youth discordant for MDD, or are there studies that examine neurocognitive functioning in unaffected first‐degree relatives longitudinally. Premorbid studies are similarly equivocal, with some evidence that lower premorbid IQ or executive functioning is associated with a mildly increased risk for MDD (David et al., 2008; Evans et al., 2016; Zammit et al., 2004), whereas other studies found no such link (Han et al., 2016; Meyer et al., 2004). Furthermore, caution is needed in interpreting the relationship between premorbid neurocognitive impairment and onset of MDD as research shows that a family history of psychiatric disorder (including mood disorders) is associated with small, but significant neurocognitive impairment (McGrath et al., 2014). This suggests that there are shared genetic factors associated with psychiatric disorder and neurocognitive impairment, rather than neurocognitive impairment being an independent risk factor (McGrath et al., 2014). Future studies that examine neurocognition as a trait risk for MDD need to take into account family history of MDD (and other psychiatric disorders).

The presence and extent of state‐based impairments in youth depression also remains unclear. Cross‐sectional studies of current MDD have shown impaired neurocognition, particularly in the domains of working memory and processing speed (Baune et al., 2014), but state effects cannot be teased apart from trait‐ and scare‐based impairments because there is no follow‐up assessment when symptoms have resolved. Similarly, although some cross‐sectional studies of youth in remission from MDD have shown evidence of impaired executive functioning (Kyte et al., 2005; Smith et al., 2006), again, it remains uncertain whether the impairments are trait‐, scar‐, or residual state‐based impairments, given participants in these studies had experienced a depressive episode within months of being tested and there was no assessment prior to remission (Kyte et al., 2005; Smith et al., 2006).

Prospective longitudinal studies that assess neurocognition prior to, during, and after initial and subsequent episodes of major depression are the most definitive for informing the type of neurocognitive patterns observed in MDD. This is particularly the case for trait‐ versus scar‐related impairments. A growing number of longitudinal studies with repeated neurocognitive assessment have been conducted in young people (Bloch et al., 2015; Peters et al., 2015; Schmid & Hammar, 2013), but only two have assessed neurocognition prior to the onset of MDD (Beaujean et al., 2013; Vijayakumar et al., 2016). Longitudinal studies have primarily focused on executive functioning due to the developmental relevance of this domain in adolescence and young adulthood (Anderson et al., 2001), as well as the established neurobiological and psychological links between executive functions and depression (Snyder, 2013). The longitudinal studies without a premorbid neurocognitive assessment have suggested mild trait‐related neurocognitive impairments in youth with MDD because the impairments remained relatively stable and unrelated to illness severity (Bloch et al., 2015; Peters et al., 2015; Schmid & Hammar, 2013). In contrast, the two studies with premorbid assessment did not support the trait model (Beaujean et al., 2013; Vijayakumar et al., 2016). These two latter studies by Beaujean et al. (2013) and Vijayakumar et al. (2016) suggested that MDD experienced during adolescence may be associated with neurocognitive scarring (specifically attenuated neurocognitive development). Importantly, these studies had sufficiently long follow‐up periods (ranging from 4 to 8 years) to enable the detection of possible scarring effects of MDD, whereas the other longitudinal studies only had follow‐ups of only weeks to months (Bloch et al., 2015; Peters et al., 2015; Schmid & Hammar, 2013), which is likely an insufficient time period for a scarring effect to be observed, particularly in adolescents who are still developing. As discussed earlier it may be that scar‐related neurocognitive impairments in youth may not become clearly evident until the young person has passed through the critical neurodevelopment that occurs during adolescence. Whereas the examination of neurocognitive functioning prior to the first onset of depressive symptoms and full‐threshold MDD remains a significant research challenge, this must be a priority for definitive findings regarding progressive neurocognitive scarring as a consequence of the illness. The studies by Beaujean et al. (2013) and Vijayakumar et al. (2016) provide a positive first step in achieving this.

What emerged from examination of this literature was the importance of taking into consideration factors that may confound the neurocognitive findings. Particularly, age of onset and pubertal stage seem to be important considerations in the severity of neurocognitive impairments early in the course of illness. Two reviews showed that older age of onset is associated with poorer neurocognition (Bora et al., 2013; Lee et al., 2012) and indicated that neurocognitive impairment in older people with MDD may have a different underlying mechanism. These findings highlight the importance of investigating youth populations separately and to take into account developmental factors, such as pubertal stage. The important moderating role of factors such as age of onset, puberty, IQ, family history, gender, medication status, duration of illness, and number of episodes need to be factored into the designs of future studies. A limitation of many extant studies in youth MDD is that neurocognitive assessment was limited to a small number of domains. Gleaning from the literature, it may be that state‐, trait‐, and scar‐based impairments occur differentially in different neurocognitive domains. For example, attention (i.e., concentration, working memory) and processing speed domains may be more effected by clinical state (in line with the clinical symptoms of depression) (Bloch et al., 2015; Lee et al., 2012), whereas higher‐order executive functions may be more permanently impacted (Belleau et al., 2013; Evans et al., 2016; Vijayakumar et al., 2016). It is recommended that future studies examine a broad range of neurocognitive domains to test this possibility.

A clinical staging heuristic has been proposed as a potentially useful framework for characterizing individuals in the early stages of MDD and providing the opportunity for early intervention (Hetrick et al., 2008; McGorry et al., 2007). Neurocognition has been suggested as a possible important biomarker in the staging model broadly, although research on the staging of neurocognition in MDD has received limited investigation (Lin, Reniers, & Wood, 2013; McGorry et al., 2014). We hope this overview is a catalyst for neurocognition to be an important consideration in this emerging research arena. As highlighted in this overview, the question remains open as to whether progressive scar‐related neurocognitive impairments are a feature of MDD that emerges in youth. Future longitudinal studies commencing before MDD onset, which include healthy comparison groups, large sample sizes, and multiple longer‐term follow‐up assessments covering the full range of neurocognitive functions will be critically informative regarding progressive neurocognitive scarring in MDD (Hasselbalch et al., 2011; McClintock, Husain, Greer, & Cullum, 2010; McDermott & Ebmeier, 2009; Snyder, 2013). Ultimately, further research on the nature of neurocognitive functioning in MDD will have important implications for clinical formulation and treatment, with the ultimate goal of improving prevention and early intervention efforts.

## Funding Information

The University of Melbourne (Grant/Award Number: ‘Ronald Philip Griffiths Fellowship’).

## Conflict of Interests

None declared.

## Endnote

1 Evidence for these latter two study designs are beyond the scope of this article as they do not focus specifically on MDD.
